# Bifurcation control of gait transition in insect locomotion

**DOI:** 10.1186/1471-2202-15-S1-P183

**Published:** 2014-07-21

**Authors:** William Barnett, Gennady Cymbalyuk

**Affiliations:** 1 Neuroscience Institute, Georgia State University, Atlanta, GA, 30302, USA

## 

Insect locomotion presents a type of pattern adaptation in which the phase relations change with the speed of walking. In forward walking, the protraction of legs progresses sequentially one after the other from posterior to anterior: a metachronal wave gait. The duration of protraction is roughly invariant to the speed of locomotion, and the duration of retraction is linearly dependent on the step period [1]. These constraints determine phase relations in a gait over a range of speeds.

We described a model of insect locomotion employing the cornerstone Shilnikov bifurcation [2,3]. This bifurcation generates mechanisms that control burst duration and interburst interval in endogenous bursting and the duration of pulse-triggered bursts in endogenously silent neurons [3,4]. We suggest that the mechanism describing the stereotypical burst responses of silent neurons explains smooth transitions between gaits. The burst duration is controlled by the half-activation voltage of a potassium current (-θ_K2_).

**Figure 1:**
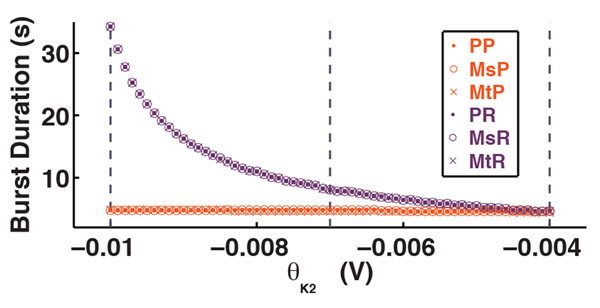
The dependence of the CPG temporal characteristics on the retractor θ_K2_ describes the transition from metachronal wave to tripod gait.

In the model, each leg was controlled by one oscillator consisting of two mutually inhibitory interneurons: protraction and retraction interneurons. The model central pattern generator (CPG) contains three coupled oscillators: PP-PR, MsP-MsR, MtP-MtR labeling protractor and retractor interneurons each for the prothoracic, mesothoracic, and metathoracic segments, respectively. The bifurcation-generated mechanisms make quantitative predictions on the CPG activity. The duration of the burst was governed by the inverse-square-root law (Figure 1). The burst duration grows linear with the number of spikes per burst in retractor interneurons.

The burst duration of the retractor interneuron determined the period of the network. The retractor burst duration determined what type of gait was exhibited by network activity. As such, we were able to control the smooth transition from metachronal wave to tripod gait. While the duty cycle of retractor interneurons was greater than 50%, we observed a gait comprised of metachronal progression of bursts from posterior to anterior. When the duty cycle became 50%, we observed the tripod gait, where the activity in the prothoracic and metathoracic protractor interneurons was synchronous.

In conclusion, we constructed a locomotor CPG model using a mechanism generated by the cornerstone bifurcation. This mechanism controls the duration of pulse-triggered bursts in endogenously silent neurons and governs a smooth transition from a metachronal gait to a tripod gait.
